# Approximating a Giving Up Smoking Dynamic on Adolescent Nicotine Dependence in Fractional Order

**DOI:** 10.1371/journal.pone.0103617

**Published:** 2016-04-22

**Authors:** Anwar Zeb, Gul Zaman, Vedat Suat ERTURK, Baha Alzalg, Faisal Yousafzai, Madad Khan

**Affiliations:** 1 Department of Mathematics, COMSATS Institute of Information Technology, Abbottabad, KPK, Pakistan; 2 Department of Mathematics, University of Malakand, Chakdara Dir (Lower), Khyber Pakhtunkhawa, Pakistan; 3 Department of Mathematics, Faculty of Arts and Sciences, Ondokuz Mayis University, 55139, Samsun, Turkey; 4 Department of Business Administration and Accounting, Buraimi University College, Al-Buraimi, Oman; 5 Department of Mathematics, COMSATS Institute of Information Technology Attack, Pakistan; H. Lee Moffitt Cancer Center & Research Institute, UNITED STATES

## Abstract

In this work, we consider giving up smoking dynamic on adolescent nicotine dependence. First, we use the Caputo derivative to develop the model in fractional order. Then we apply two different numerical methods to compute accurate approximate solutions of this new model in fractional order and compare their results. In order to do this, we consider the generalized Euler method (GEM) and multi-step generalized differential transform method (MSGDTM). We also show the unique positive solution for this model and present numerical results graphically.

## Introduction

Mathematical modeling is a tool which have a lot of applications in applied sciences that require a deep study for the different verities of methods used in applied mathematics. Smoking is the prodigious cause of many diseases specially of different type of cancers. As cigarette smoke contains over forty thousands dangerous chemicals, which cause harmful infections to human health. The life of smokers is ten to thirteen years shorter than that of non smokers and at the rat of 1:2 of smokers die from diseases launched by the cigarette smoking. Besides, smokers have 70% more chances of heart attack than non smokers. The incidence rate of lung cancer is 10% high in smokers. To secure the life expectancy every scientist, doctor and mathematician try to control smoking. Mathematicians try to make different smoking models for the best representation of cigarette smoking phenomena. So several authors proposed different smoking models, for example see the first model presented by Castillo-Garsow et al., [1] in which they studied different classes of smokers (potential smokers (P), smokers (S), and quit smokers (Q)). Then Sharami et al., [2] modified the model presented in [1] and introduced a new class named chain smokers. In their work, they presented the development and public health impact of smoking related diseases. Zeb et al., [3] presented the square-root dynamics of a giving up smoking model. In this work they discussed local and global stability of the model and its general solutions in which the interaction of occasional and potential smokers occur. Nowadays, every researcher tries to establish different epidemic models in fractional order. Perhaps, the reality of nature could be better translated by fractional calculus. Fractional calculus is used in many fields of sciences [4–15]. In this paper, we present the fractional order derivative and find analytic numeric solution of model presented in [16], and the model is as follow:

$$\begin{array}{ccc} \frac{d}{dt}P\left(t\right) & = & b_{1}\Lambda -\beta_{1}\frac{P(S_{1}+\Psi S_{2})}{N}-\left(d_{1}+\mu \right)P,\\ \frac{d}{dt}S_{1}\left(t\right) & = & b_{2}\Lambda +\beta_{1}\frac{P(S_{1}+\Psi S_{2})}{N}+\alpha \left(1-\sigma \right)Q_{t}-\left(\beta_{2}+\gamma_{1}+u_{1}+d_{2}+\mu \right)S_{1},\\ \frac{d}{dt}S_{2}\left(t\right) & = & b_{3}\Lambda +\beta_{2}S_{1}+\alpha \sigma Q_{t}-\left(\gamma_{2}+u_{2}+d_{3}+\mu \right)S_{1},\\ \frac{d}{dt}Q_{t}\left(t\right) & = & b_{4}\Lambda +\left(\gamma_{1}+u_{1}\right)S_{1}+\left(\gamma_{2}+u_{2}\right)S_{2}-\left(\gamma_{3}+\alpha +d_{4}+\mu \right)Q_{t},\\ \frac{d}{dt}Q_{p}\left(t\right) & = & b_{5}\Lambda +\gamma_{3}Q_{t}-\left(\mu +d_{5}\right)Q_{p}, \end{array}$$ (1)

under the initial conditions:

$$\begin{array}{c} P\left(0\right)=e_{1},\;\;S_{1}\left(0\right)=e_{2},\;\;S_{2}\left(0\right)=e_{3},\;\;Q_{t}\left(0\right)=e_{4},\;\;Q_{p}\left(0\right)=e_{5}, \end{array}$$ (2)

where *P*, *S*_1_, *S*_2_, *Q*_*t*_ and *Q*_*p*_ all functions of *t* denote the numbers of potential, occasional and quit smokers. Here Λ is number of incoming population per unit time, *b*_*i*_ is incoming rate per unit time *i* = 1, 2, 3, 4, 5 $\left(b=\sum_{i=1}^{5}b_{i}=1\right)$: Average rate of incoming for a year, *d*_*i*_: Outgoing per unit time *i* = 1, 2, 3, 4, 5, *μ* is natural death rate or death related to other diseases not smoking and *d* represents death rate of all classes caused by smoking diseases.

By establish fractional order derivative into the model [16], we obtain the following fractional order model:

$$\begin{array}{ccc} D_{t}^{\alpha }P\left(t\right) & = & b_{1}\Lambda -\beta_{1}\frac{P(S_{1}+\Psi S_{2})}{N}-\left(d_{1}+\mu \right)P,\\ D_{t}^{\alpha }S_{1}\left(t\right) & = & b_{2}\Lambda +\beta_{1}\frac{P(S_{1}+\Psi S_{2})}{N}+\alpha \left(1-\sigma \right)Q_{t}-\left(\beta_{2}+\gamma_{1}+u_{1}+d_{2}+\mu \right)S_{1},\\ D_{t}^{\alpha }S_{2}\left(t\right) & = & b_{3}\Lambda +\beta_{2}S_{1}+\alpha \sigma Q_{t}-\left(\gamma_{2}+u_{2}+d_{3}+\mu \right)S_{1},\\ D_{t}^{\alpha }Q_{t}\left(t\right) & = & b_{4}\Lambda +\left(\gamma_{1}+u_{1}\right)S_{1}+\left(\gamma_{2}+u_{2}\right)S_{2}-\left(\gamma_{3}+\alpha +d_{4}+\mu \right)Q_{t},\\ D_{t}^{\alpha }Q_{p}\left(t\right) & = & b_{5}\Lambda +\gamma_{3}Q_{t}-\left(\mu +d_{5}\right)Q_{p}. \end{array}$$ (3)

Here we consider the Caputo sense fractional derivative and *α* is the order of the fractional time-derivative, subject to the initial conditions given in Eq (2). In the present paper, we take in account the model presented in [16]. First, we will use the Caputo derivative to develop the model in fractional order. Then we will apply two different numerical methods to compute accurate approximate solutions of this new model in fractional order and compare their results. In order to do this, we consider generalized Euler method (GEM) and the multi-step generalized differential transform method (MSGDTM). We also show the unique positive solution for this model and present numerical results graphically.

This paper is organized as follows. In Section 2, we present positivity of the new model. The idea of GEM and MSGDTM for solution of the proposed model are presented shortly in Sections 3. Numerical simulation results graphically of GEM and MSGDTM are presented in Section 4 with comparisons with the results of Runge-Kutta method (RKM). The conclusion is given in Section 5. Appendix is devoted to present some basic definitions and results which is needed in this work are given in the supporting file.

## 1 Non-negative Solutions

Let $R_{+}^{5}=\left\{X\in R^{5}:X\geq 0\right\}$ and *X* = (*P*, *S*_1_, *S*_2_, *Q*_*t*_, *Q*_*p*_)^*T*^.

We need lemma [Generalized Mean Value Theorem] [17] that help us in the proof of subsequent theorem.

**Theorem 1** *If* *P* > 0, *S*_1_ > 0, *S*_2_ > 0, *Q*_*t*_ > 0, *Q*_*p*_ > 0, *all at* (0) *the solutions of P*, *S*_1_, *S*_2_, *Q*_*t*_, *Q*_*p*_ *at* (*t*) *of the system* Eq (3), *are positive for all t* > 0.

**Proof 1** *If the above condition does not satisfied, then at least one of the individuals may be negative. Then the individuals will satisfy one of the following conditions*.

1. *There exists a first time t*_1_
  $$\begin{array}{c} P\left(t_{1}\right)=0,\;\;\overset{´}{P}\left(t_{1}\right)=0,\;\;S_{1}\left(t\right)\geq 0,\;\;S_{2}\left(t\right)\geq 0,\;\;Q_{t}\left(t\right)\geq 0,\;\;Q_{p}\left(t\right)\geq 0, \end{array}$$
  $$\begin{array}{c} \;\;0\leq t\leq t_{1}. \end{array}$$
  *But* $\overset{´}{P}\left(t_{1}\right){|}_{P(t_{1})=0}=b_{1}\Lambda \geq 0$, *which is contradiction to the above supposition*.
2. *There exists a first time t*_2_
  $$\begin{array}{c} S_{1}\left(t_{2}\right)=0,\;\;\overset{´}{S_{1}}\left(t_{2}\right)=0,\;\;P\left(t\right)\geq 0,\;\;S_{2}\left(t\right)\geq 0,\;\;Q_{t}\left(t\right)\geq 0,\;\;Q_{p}\left(t\right)\geq 0, \end{array}$$
  $$\begin{array}{c} \;\;0\leq t\leq t_{2}. \end{array}$$
  *But* ${\overset{´}{S}}_{1}\left(t_{2}\right){|}_{S_{1}\left(t_{2}\right)=0}=b_{2}\Lambda +\beta_{2}\frac{P(\Psi S_{2})}{N}+\alpha \left(1-\sigma \right)Q_{t}\geq 0,$, *which is contradiction to the above supposition*.
3. *There exists a first time t*_3_
  $$\begin{array}{c} S_{2}\left(t_{3}\right)=0,\;\;\overset{´}{S_{2}}\left(t_{3}\right)=0,\;\;P\left(t\right)\geq 0,\;\;S_{1}\left(t\right)\geq 0,\;\;Q_{t}\left(t\right)\geq 0,\;\;Q_{p}\left(t\right)\geq 0, \end{array}$$
  $$\begin{array}{c} \;\;0\leq t\leq t_{3}. \end{array}$$
  *But* ${\overset{´}{S}}_{2}\left(t_{3}\right){|}_{S_{2}\left(t_{3}\right)=0}=b_{3}\Lambda +\beta_{2}S_{1}+\alpha \sigma Q_{t}\geq 0,$, *which is contradiction to the above supposition*.
4. *There exists a first time t*_4_
  $$\begin{array}{c} P\left(t_{4}\right)=0,\;\;\overset{´}{Q_{t}}\left(t_{4}\right)=0,\;\;P\left(t\right)\geq 0,\;\;S_{1}\left(t\right)\geq 0,\;\;S_{2}\left(t\right)\geq 0,\;\;Q_{p}\left(t\right)\geq 0, \end{array}$$
  $$\begin{array}{c} \;\;0\leq t\leq t_{4}. \end{array}$$
  *But* ${\overset{´}{Q}}_{t}\left(t_{4}\right){|}_{Q_{t}\left(t_{4}\right)=0}=b_{4}\Lambda +\left(\gamma_{1}+u_{1}\right)S_{1}+\left(\gamma_{2}+u_{2}\right)S_{2}\geq 0,$, *which is contradiction to the above supposition*.
5. *There exists a first time t*_5_
  $$\begin{array}{c} Q_{p}\left(t_{5}\right)=0,\;\;\overset{´}{Q_{p}}\left(t_{5}\right)=0,\;\;P\left(t\right)\geq 0,\;\;S_{1}\left(t\right)\geq 0,\;\;S_{2}\left(t\right)\geq 0,\;\;Q_{t}\left(t\right)\geq 0, \end{array}$$
  $$\begin{array}{c} \;\;0\leq t\leq t_{5}. \end{array}$$
  *But* ${\overset{´}{Q}}_{p}\left(t_{5}\right){|}_{Q_{p}\left(t_{5}\right)=0}=b_{5}\Lambda +\gamma_{3}Q_{t}\geq 0$, *which is contradiction to the above supposition*.

## 2 Generalized Euler Method (GEM) and Multistep Generalized Differential Transform Method (MSGDTM)

Generally, it is impossible to determine the analytic solution of nonlinear differential equations. However, different authors used different methods for the approximate analytic and numeric solutions of nonlinear differential equations [7–11]. But all these methods are applicable for short time interval. So we have in mind to find the approximate solutions of problems Eqs (2) and (3) via the GEM and MSGDTM. The GEM is derived to get numerical solution of initial value problems with Caputo derivatives by Odibat et al., [11]. This method is a generalization of the classical Euler’s method for detail see [11]. Also in this section, we use the MSGDTM that to find numerical solution of the system Eq (3) of fractional order differential equations and the detail analysis of this method is found in [12]. The basics steps of the GDTM can be found in [12–15]. In this paper, we are not going in detail of these methods but we use them only for the numerical solution of the system Eq (3). Applying the MSGDTM, we obtain the following system:

$$\begin{array}{ccc} P(k+1) & = & \frac{\Gamma (\alpha k+1)}{\Gamma ((\alpha k+1)+1)}\left(b_{1}\Lambda -\beta_{1}PS_{1}S_{2}N\left(k\right)-\left(\mu +d_{1}\right)P\left(k\right)\right),\\ S_{1}\left(k+1\right) & = & \frac{\Gamma (\alpha k+1)}{\Gamma ((\alpha k+1)+1)}\left(b_{2}\Lambda +\beta_{1}PS_{1}S_{2}N\left(k\right)-\left(\gamma_{1}+\mu \right)S_{1}\left(k\right)\right),\\ S_{2}\left(k+1\right) & = & \frac{\Gamma (\alpha k+1)}{\Gamma ((\alpha k+1)+1)}\left(b_{3}\Lambda +\beta_{2}S_{1}\left(k\right)+\alpha \sigma Q_{t}-\left(\gamma_{2}+u_{2}+\mu +d_{3}\right)S_{2}\left(k\right)\right),\\ Q_{t}\left(k+1\right) & = & \frac{\Gamma (\alpha k+1)}{\Gamma ((\alpha k+1)+1)}\left(b_{4}\Lambda +\left(\gamma_{1}+u_{1}\right)S_{1}\left(k\right)+\left(\gamma_{2}+u_{2}\right)S_{2}\left(k\right)-\left(\gamma_{3}+\alpha +\mu +d_{4}\right)Q_{t}\left(k\right)\right),\\ Q_{p}\left(k+1\right) & = & \frac{\Gamma (\alpha k+1)}{\Gamma ((\alpha k+1)+1)}\left(b_{5}\Lambda +\gamma_{3}Q_{t}\left(k\right)-\left(\mu +d_{5}\right)Q_{p}\left(k\right)\right). \end{array}$$

Here, *P*(*k*), *S*_1_(*k*), *S*_2_(*k*), *Q*_*t*_(*k*) and *Q*_*p*_(*k*) are the differential transformation of *P*(*t*), *S*_1_(*t*), *S*_2_(*t*), *Q*_*t*_(*t*) and *Q*_*p*_(*t*), respectively. Also, *PS*_1_ *S*_2_ *N*(*k*) is the differential transformation of function

$$\begin{array}{c} PS_{1}S_{2}N\left(k\right)=\frac{P(S_{1}\left(t\right)+\Psi S_{2}\left(t\right))}{N(t)} \end{array}$$

and defined as follows:

$$\begin{array}{c} PS_{1}S_{2}N\left(k\right)=\frac{1}{N(0)}\left[\sum_{s=0}^{k}P\left(S_{1}\left(k-s\right)+\Psi S_{2}\left(k-s\right)\right)-\sum_{s=0}^{k-1}PS_{1}S_{2}N\left(s\right)N\left(k-s\right)\right]. \end{array}$$

In next section, we will present the numerical results.

## 3 Numerical and Simulation Results

We applied the GEM and MSGDTM to solve the system Eq (1) for *α* = 1. In order to demonstrate the effectiveness of these two methods as an approximate tool for solving the nonlinear system of fractional differential Eq (3) for larger time *t*, we apply these two methods on the interval [0, 30]. It is to be noted that GEM results are obtained for *h* = 0.00001 while the MSGDTM results are obtained when *K* = 10 and *M* = 30000 and RKM for *h* = 0.001. All the results are calculated by using computer algebra package Mathematica and in this paper, we show only the graphically obtained results.

**Fig 1:**
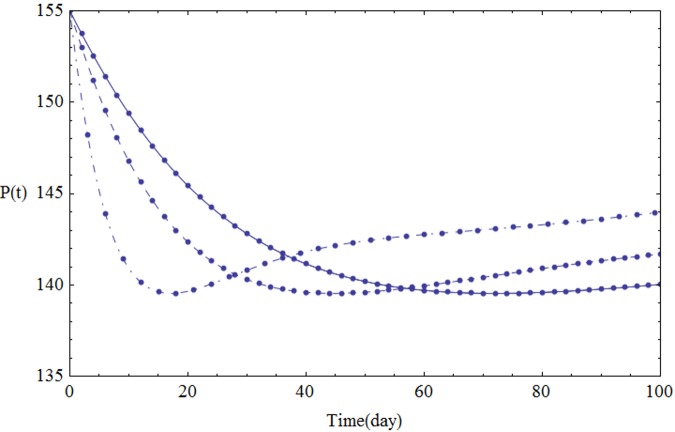
The population of *P*(*t*) versus t: *α* = 1.0 (solid line), *α* = 0.95 (dashed line) and *α* = 0.85 (dot-dashed line).

**Fig 2:**
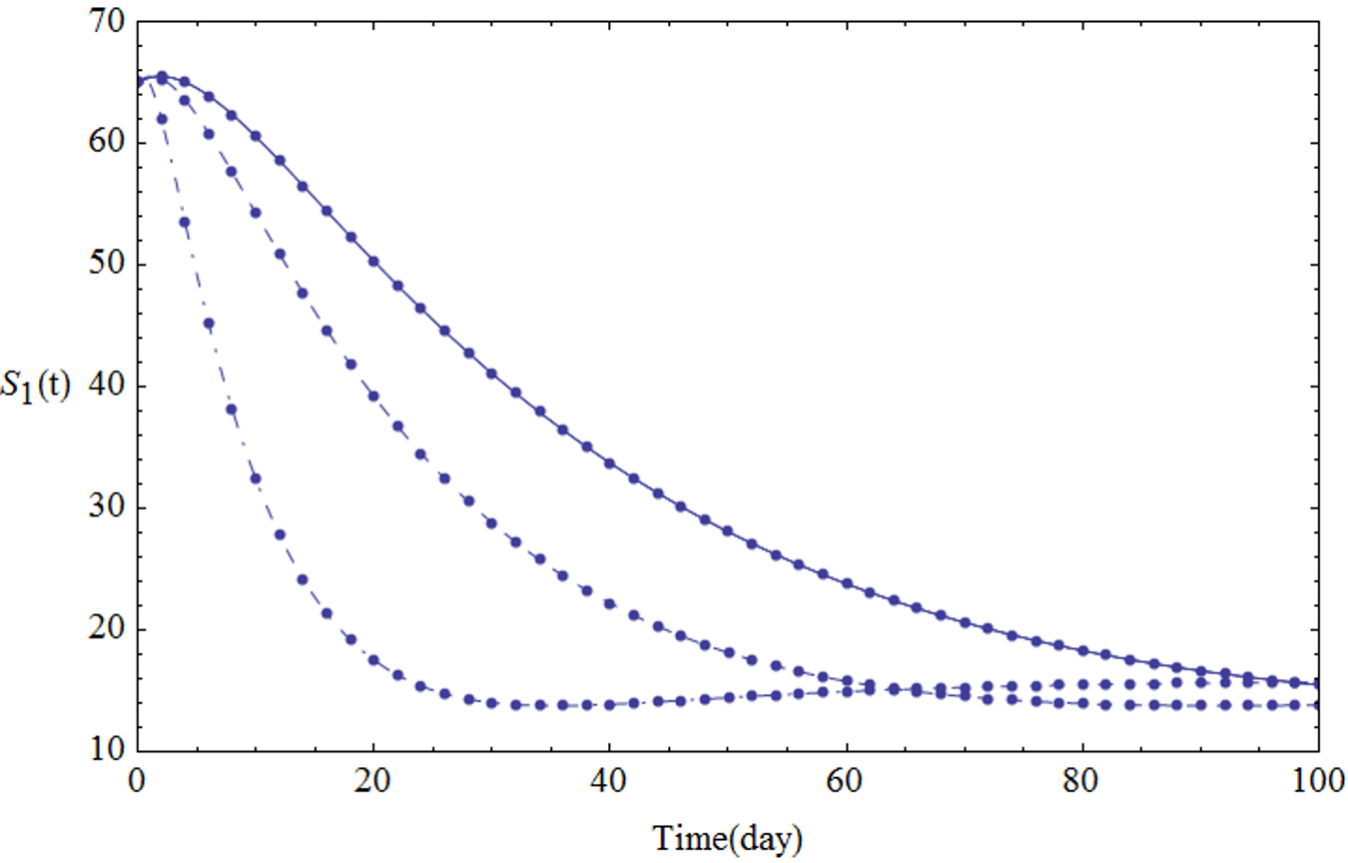
The population of *S*_1_(*t*) versus t: *α* = 1.0 (solid line), *α* = 0.95 (dashed line) and *α* = 0.85 (dot-dashed line).

**Fig 3:**
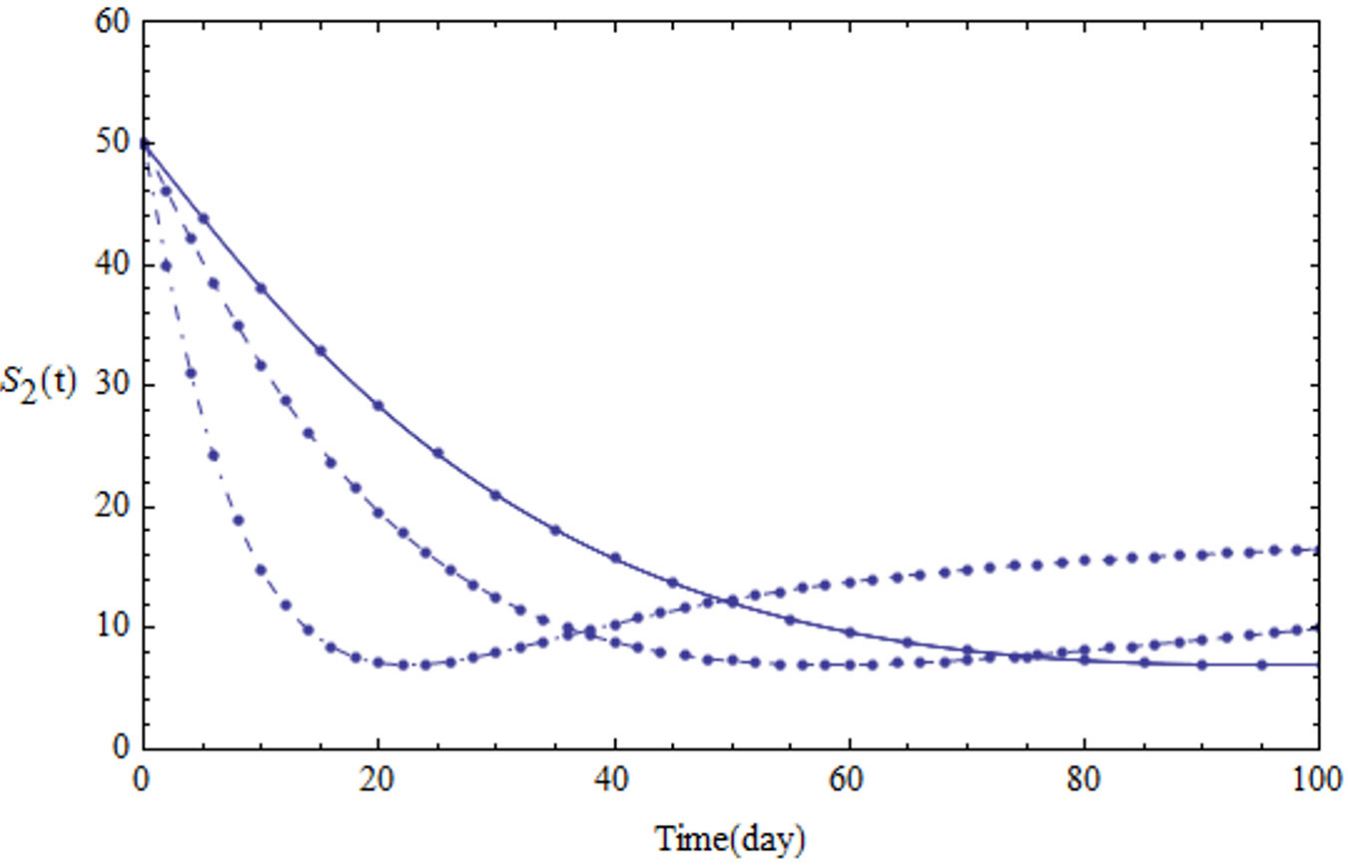
The population of *S*_3_(*t*) versus t: *α* = 1.0 (solid line), *α* = 0.95 (dashed line) and *α* = 0.85 (dot-dashed line).

**Fig 4:**
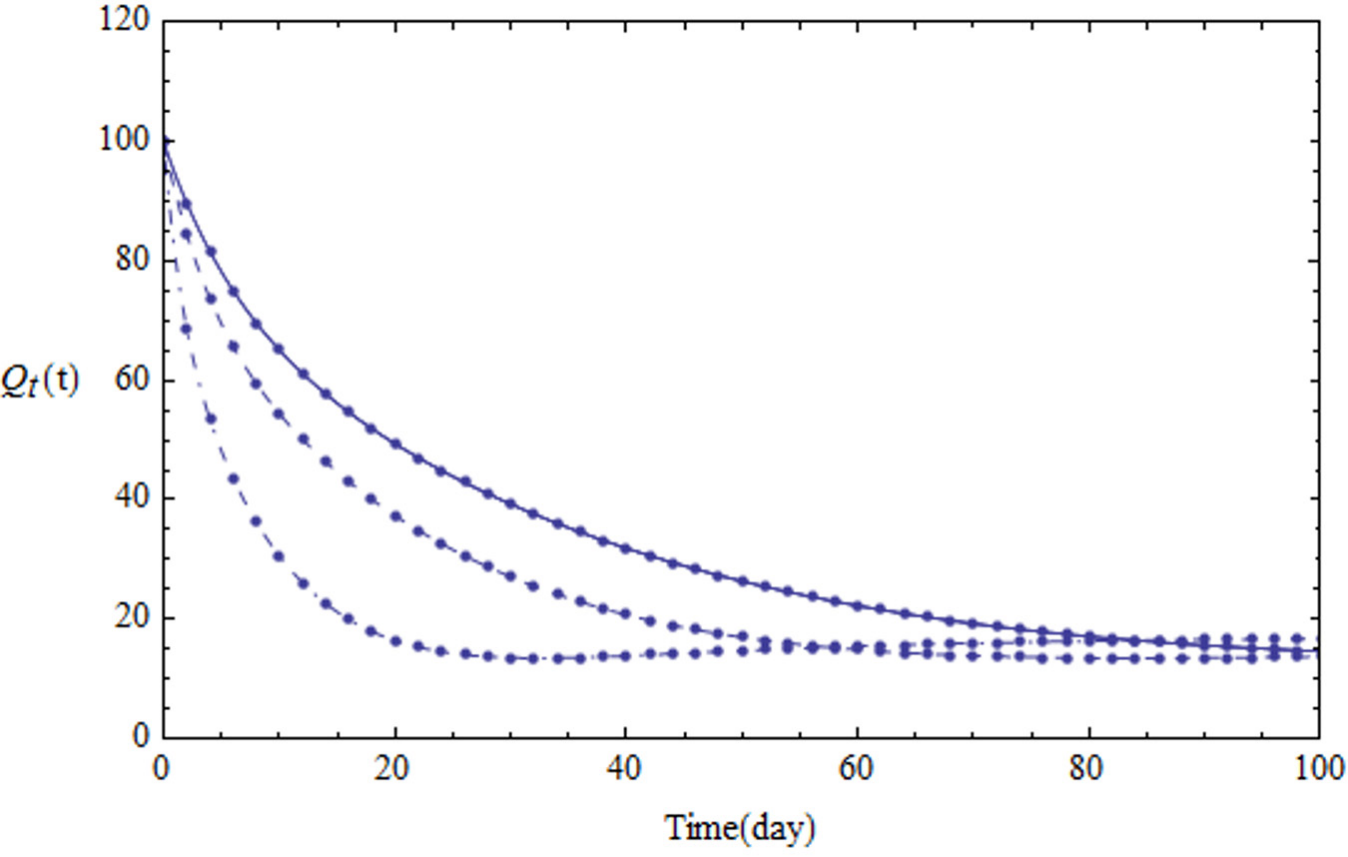
The population of *Q*_*t*_(*t*) versus t: *α* = 1.0 (solid line), *α* = 0.95 (dashed line) and *α* = 0.85 (dot-dashed line).

**Fig 5:**
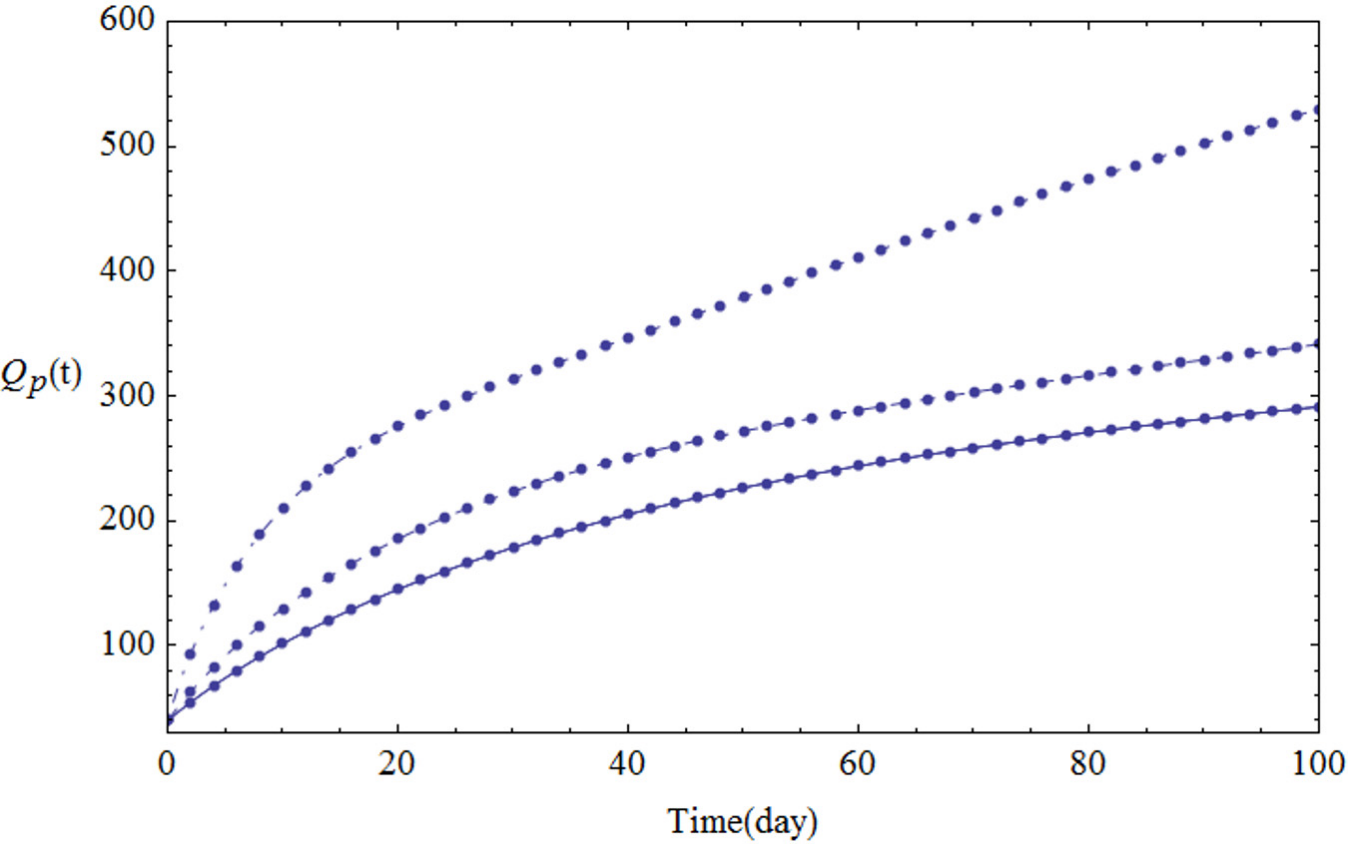
The population of *Q*_*t*_(*t*) versus t: *α* = 1.0 (solid line), *α* = 0.95 (dashed line) and *α* = 0.85 (dot-dashed line).

We assume the parameters of the system Eq (1) shown in Table 1. Figs 1–5 show the approximate solutions for *P*(*t*), *S*_1_(*t*), *S*_2_(*t*), *Q*_*t*_(*t*) and *Q*_*p*_(*t*) obtained for different values of *α* using GEM. From the graphical results given in Figs 1–5, it can be seen that the results obtained using GEM match the results of Runge-Kutta for integer case(*α* = 1,) very well when *α* = 1, which implies that GEM can predict the behavior of these variables accurately for the region under consideration.

**Table 1:** The parameters of system Eq (1).

Parameter	Range	Value
Λ	2 per week	2
*u*_1_	*u*_1_ ∈ (0, ∞)	0.047
*b*_*i*_(*i* = 1, …, 5)	*b*_*i*_ ∈ (0,1)	0.2
*u*_2_	*u*_2_ ∈ (0, ∞)	0.023
*d*_*i*_(*i* = 1, …, 5)	*d*_*i*_ ∈ [0, 1)	0
*γ*_1_	*γ*_1_ ∈ (0, ∞)	0.025
*γ*_2_	*γ*_1_ ∈ (0, ∞)	0.012
*γ*_3_	*γ*_1_ ∈ (0, ∞)	0.074
*μ*	520 weeks	0.0019
*β*_2_	*β*_2_ ∈ (0, ∞)	0.012
∝	∝ ∈ (0, 1)	0.05
Ψ	Ψ ≥ 1	2

## Discussion

In this paper, we used Generalized Euler method (GEM) and multi-step generalized differential transform method (MSGDTM) as a reasonable basis for studying the dynamics of a new giving up smoking model and modified the integer order model Eq (1) into a fractional-order model Eq (3). The results obtained show that when *α* → 1 the solutions of fractional model, {*P*}_*α*_(*t*), {*S*_1_}_*α*_(*t*), {*S*_2_}_*α*_(*t*), {*Q*_*t*_}_*α*_(*t*) and {*Q*_*p*_}_*α*_(*t*), reduce to the standard solutions *P*(*t*), *S*_1_(*t*), *S*_2_(*t*), *Q*_*t*_(*t*) and *Q*_*p*_(*t*). Finally, the recent appearance of fractional differential equations as models in some fields of Science and Engineering makes it necessary to investigate analytical and numerical methods of solution for such equations. The present success of the proposed methods for the considered model verifies that it is a useful tool for these kind of models in Science and Engineering.

## Appendix

### Basics of Fractional Calculus

In this appendix, we give some basic definitions and properties of the fractional calculus theory which are used further in [4–6, 17, 18].

**Definition 1** *A real function f*(*x*)(*x* > 0) *is said to be in the space C*_*α*_(*α* ∈ *R*) *iff it can be written as f*(*x*) = *x*^*p*^ *f*_1_(*x*) *for some p* > *α* *where f*_1_(*x*) *is continuous in* [0, ∞), *and it is said to be in the space* $C_{\alpha }^{m}$ *if f*^(*m*)^ ∈ *C*_*α*_, *m* ∈ *N*.

**Definition 2** *The Riemann–Liouville integral operator of order α* > 0 *with a* ≥ 0 *is defined as*

$$\begin{array}{c} \left(J_{a}^{\alpha }f\right)\left(x\right)=\frac{1}{\Gamma (\alpha )}\int_{a}^{x}{\left(x-t\right)}^{\alpha -1}f\left(t\right)dt,\;\;x>a, \end{array}$$ (4)

$$\begin{array}{c} \left(J_{a}^{0}f\right)\left(x\right)=f\left(x\right). \end{array}$$ (5)

Properties of this operator can be found in [4]. We only need here the following:

For *f* ∈ *C*_*α*_, *α*, *β* > 0, *a* ≥ 0, *c* ∈ *R* and *γ* > −1, we have

$$\begin{array}{c} \left(J_{a}^{\alpha }J_{a}^{\beta }f\right)\left(x\right)=\left(J_{a}^{\beta }J_{a}^{\alpha }f\right)\left(x\right)=\left(J_{a}^{\alpha +\beta }f\right)\left(x\right) \end{array}$$ (6)

$$\begin{array}{c} J_{a}^{\alpha }x^{\gamma }=\frac{x^{\gamma +\alpha }}{\Gamma (\alpha )}B_{\frac{x-a}{x}}\left(\alpha ,\gamma +1\right), \end{array}$$ (7)

where *B*_*τ*_(*α*, *γ*+1) is the incomplete beta function which is defined as

$$\begin{array}{c} B_{\tau }\left(\alpha ,\gamma +1\right)=\int_{0}^{\tau }t^{\alpha -1}{\left(1-t\right)}^{\gamma }dt, \end{array}$$ (8)

$$\begin{array}{c} J_{a}^{\alpha }e^{cx}=e^{ac}{\left(x-a\right)}^{\alpha }\sum_{k=0}^{\infty }\frac{{\left[c(x-a)\right]}^{k}}{\Gamma (\alpha +k+1)}. \end{array}$$ (9)

The Riemann-Liouville derivative has certain disadvantages when trying to model real-world phenomena with fractional differential equations. For example, the Riemann-Liouville derivative of a constant is not zero. In addition, if an arbitrary function is a constant at the origin, its fractional derivation has a singularity at the origin for instant exponential and Mittag-Leffler functions. Theses disadvantages reduce the field of application of the Riemann-Liouville fractional derivative. One of the great advantages of the Caputo fractional derivative is that it allows traditional initial and boundary conditions to be included in the formulation of the problem [18]. Therefore, we shall introduce a modified fractional differential operator $D_{a}^{\alpha }$ proposed by Caputo in his work on the theory of viscoelasticity.

**Definition 3** *The Caputo fractional derivative of f*(*x*) *of order* *α* > 0 *with a* ≥ 0 *is defined as*

$$\begin{array}{c} \left(D_{a}^{\alpha }f\right)\left(x\right)=\left(J_{a}^{m-\alpha }f^{\left(m\right)}\right)\left(x\right)=\frac{1}{\Gamma (m-a)}\int_{a}^{x}\frac{f^{\left(m\right)}\left(t\right)}{{\left(x-t\right)}^{\alpha +1-m}}dt, \end{array}$$ (10)

The Caputo fractional derivative was investigated by many authors,

$$\begin{array}{c} \left(J_{a}^{\alpha }D_{a}^{\alpha }f\right)\left(x\right)=J^{m}D^{m}f\left(x\right)=f\left(x\right)-\sum_{k=0}^{m-1}f^{\left(k\right)}\left(a\right)\frac{{\left(x-a\right)}^{k}}{k!}. \end{array}$$ (11)

**Theorem 2 (Generalized Taylor’s formula)** [17] *Suppose that* $D_{a}^{k\alpha }f\left(x\right)\in C\left(a,b\right]$ *for k* = 0, 1, …, *n* + 1, *where* 0 < *α* ≤ 1. *Then we have*

$$\begin{array}{c} f\left(x\right)=\sum_{i=0}^{n}\frac{{\left(x-a\right)}^{i\alpha }}{\Gamma (i\alpha +1)}\left(D_{a}^{i\alpha }\right)\left(a\right)+\frac{\left(D_{a}^{(n+1)\alpha }f\right)\left(\xi \right)}{\Gamma ((n+1)\alpha +1)}{\left(x-a\right)}^{(n+1)\alpha }, \end{array}$$ (12)

*with a* ≤ *ξ* ≤ *x*, ∀*x* ∈ (*a*, *b*], *where* $D_{a}^{n\alpha }=\underset{n-times}{\underset{︸}{D_{a}^{\alpha }D_{a}^{\alpha }\cdots D_{a}^{\alpha }}}$.

For mathematical properties of fractional derivatives and integrals one can consult the mentioned references in this paper.
