# Correction: Approximating a Giving Up Smoking Dynamic on Adolescent Nicotine Dependence in Fractional Order

**DOI:** 10.1371/journal.pone.0155091

**Published:** 2016-05-03

**Authors:** Anwar Zeb, Gul Zaman, Vedat Suat ERTURK, Baha Alzalg, Faisal Yousafzai, Madad Khan

There is an error in affiliation 4 for author Baha Alzig. Affiliation 4 should be: Department of Mathematics, The University of Jordan, Amman 11942, Jordan.
